# Musical Meter Induces Interbrain Synchronization during Interpersonal Coordination

**DOI:** 10.1523/ENEURO.0504-21.2022

**Published:** 2022-10-26

**Authors:** Yinying Hu, Min Zhu, Yang Liu, Zixuan Wang, Xiaojun Cheng, Yafeng Pan, Yi Hu

**Affiliations:** 1 Shanghai Key Laboratory of Mental Health and Crisis Intervention, School of Psychology and Cognitive Science, East China Normal University, Shanghai 200062, China; 2 College of Emergency Management, Nanjing Tech University, Nanjing 211816, China; 3 School of Psychology, Shenzhen University, Shenzhen 518060, China; 4 Department of Psychology and Behavioral Sciences, Zhejiang University, Hangzhou 310058, China

**Keywords:** interbrain synchronization, musical meter, interpersonal coordination, fNIRS hyperscanning

## Abstract

Music induces people to coordinate with one another. Here, we conduct two experiments to examine the underlying mechanism of the interbrain synchronization (IBS) that is induced by interpersonal coordination when people are exposed to musical beat and meter. In experiment 1, brain signals at the frontal cortex were recorded simultaneously from two participants of a dyad by using functional near-infrared spectroscopy (fNIRS) hyperscanning, while each tapped their fingers to aural feedback from their partner (coordination task) or from themselves (independence task) with and without the musical meter. The results showed enhanced IBS at the left-middle frontal cortex in case of the coordination task with musical beat and meter. The IBS was significantly correlated with the participants performance in terms of coordination. In experiment 2, we further examined the IBS while the participants coordinated their behaviors in various metrical contexts, such as strong and weak meters (i.e., high/low loudness of acoustically accenting beats). The results showed that strong meters elicited higher IBS at the middle frontal cortex than weak meters. These findings reveal that the musical beat and meter can affect brain-to-brain coupling in action coordination between people, and provide insights into the interbrain mechanism underlying the effects of music on cooperation.

## Significance Statement

This study reveals enhanced interbrain synchronization approximately at the left-middle frontal cortex, during interpersonal coordination in the presence of musical beat and meter. The behavioral performance of the participants of a dyad during metrical coordination was also predicted. Further, the interbrain synchronization was modulated by the accent of the musical meter, with higher interbrain synchronization in case of strong meters than weak ones. These results suggest that music enhances interbrain synchronization to promote interpersonal coordination.

## Introduction

Music moves us to coordinate with others (Keller et al., 2014). When people get together for activities involving music, they show better interpersonal coordination than otherwise across populations (e.g., infants, children, and adults; Kirschner and Tomasello, 2010; Gerry et al., 2012; Lang et al., 2016), population sizes (e.g., pair, group; Wiltermuth and Heath, 2009; Codrons et al., 2014), and types of collaboration (e.g., intentional, unintentional; Gioia, 2006; Demos et al., 2012). However, the brain mechanism underlying the effects of music on interpersonal coordination remains incompletely understood.

Research on brain imaging has mapped distributed brain networks that are engaged in rhythmic interpersonal coordination, including the activation of the auditory cortex, parietal cortex, thalamus, and caudate in synchronized drumming (Kokal et al., 2011), and the activation of the motor cortex as well as the cerebellum in rhythmic coordination (Mayville et al., 2002). These results have been obtained from comparisons between synchronized and unsynchronized conditions, or between coordinated and baseline conditions, rather than through a direct comparison between conditions involving the presence and absence of music. Further, previous studies on neuroimaging have focused on rhythmic interpersonal coordination at the level of an individual’s brain activity. Social interactions, which include interpersonal coordination, have recently been considered as a feedback loop in a multibrain system (Kingsbury and Hong, 2020). That is, interpersonal coordination requires at least two brains to communicate with each other. Therefore, previous findings are not necessarily informative regarding the brain mechanism underlying the effect of music on interpersonal coordination.

Recent research on brain imaging has used the hyperscanning approach to simultaneously measure brain signals from two or more individuals (Montague, 2002) to explore the neural communication between the interactive partners (Dumas et al., 2010; Yun et al., 2012). A growing body of studies on interpersonal coordination based on functional near-infrared spectroscopy (fNIRS)/electroencephalogram (EEG) hyperscanning has revealed interbrain synchronization (IBS) during various forms of interpersonal coordination, such as the cooperative key-press (Cui et al., 2012), joint drawing (Cheng et al., 2022), synchronized movement (Hu et al., 2017), and action imitation (Miyata et al., 2021). Moreover, interpersonal coordination in musical contexts has been found to be accompanied by IBS, including rhythmic finger-tapping (Konvalinka et al., 2014), rhythmic arm swinging (Nozawa et al., 2019), rhythmic group-walking (Ikeda et al., 2017), groups playing drums (Liu et al., 2021), playing guitars in a quartet (Müller et al., 2018), and singing and humming (Osaka et al., 2015; Müller et al., 2019). IBS in the frontal and parietal cortices has been mapped with and without music (Cheng et al., 2019; Feng et al., 2020). It is always positively correlated with behavioral coordination (Mu et al., 2016), where this is interpreted as a correlate of behavioral or cognitive alignment (Kingsbury and Hong, 2020). Taken together, this body of research suggests that IBS plays a key role in interpersonal coordination regardless of whether the subjects are exposed to music.

Nevertheless, the diversity of IBS between conditions with and without music remains unclear. Based on evidence whereby music enhances behavioral coordination, we predicted in past work that there is greater IBS during interpersonal coordination when the subjects are exposed to music than when they are not. Specifically, we focused on the abstract temporal information of music—musical beat and meter—and hierarchically grouped downbeats and upbeats (Cooper and Meyer, 1960). The reason for this focus was that musical beat and meter play an important role in our understanding of the effects of music, especially the temporal coordination of actions. That is, the understanding of music is inseparable from that of the beat and meter, and our interaction with music is the ability to identify and move in time with every beat while accenting downbeats compared with upbeats (Dixon and Cambouropoulos, 2000; Snyder and Krumhansl, 2001).

In this study, we directly compare IBS in interpersonal coordination between metrical and no-metrical contexts, and examine how it changes in various metrical contexts (i.e., different accents/frequencies of occurrence of downbeats and upbeats). Accent and the frequency of occurrence are key regularities needed to perceive musical meter (Palmer and Krumhansl, 1990). The former is one whereby downbeats last longer, are louder and higher in pitch, or are positioned at points of change in a melody. The latter is typically displayed as a march (perceived as one-two-one …) or a waltz (perceived as one-two-three-one …). We hypothesize that there is higher IBS underlying interpersonal coordination in case of exposure to musical meter (vs no musical meter) that is enhanced when highlighting the meter. To test our hypothesis, we conducted two fNIRS hyperscanning experiments. fNIRS technology was used because of its loose constraints on measurements in relatively natural settings (Egetemeir et al., 2011). Brain signals were recorded in an extended area of the frontal cortex (including the motor and premotor areas), based on the location of the IBS in previous studies. In experiment 1, dyads of participants were asked to tap their fingers to auditory feedback from their partner or themselves after listening to meter and no-meter stimuli. We anticipated higher IBS and better behavioral coordination when the participants tapped together after listening to the metrical stimuli than to no-metrical stimuli. In experiment 2, the participants were required to tap their fingers to auditory feedback from their partner after listening to different metrical stimuli, such as meters with different accents (i.e., strong vs weak) and frequencies of occurrence (i.e., duple vs triple). We anticipated enhanced IBS and better behavioral coordination in the participants when they listened to outstanding meter stimuli (e.g., strong meters) because stronger meters are known to elicit higher brain activity and better coordination than weak meters (J.L. Chen et al., 2008).

## Experiment 1

### Materials and Methods

#### Participants

Forty graduate and undergraduate female college students (mean age = 21.820 years, range: 18–25 years) were recruited for experiment 1 in randomly matched dyads in exchange for monetary compensation. Only female participants were recruited as gender is known to have an effect in the context of music (Christenson and Peterson, 1988) and IBS (Cheng et al., 2015; M. Chen et al., 2020). Members of a given dyad had not seen or known each other before. They were right-handed, and had normal or corrected-to-normal vision and hearing. They had either not studied music or had studied it for fewer than three years. Ethics approval was obtained from the University Committee on Human Research Protection of Author University.

#### Stimuli and apparatus

The auditory stimuli consisted of 440-Hz pure tones that lasted for 660 ms. They were created by MuseScore, a free music composition and notation software (https://musescore.org/en). The auditory stimuli consisted of two types of tone sequences. One was a meter tone sequence while the other was a no-meter tone sequence. Each tone sequence lasted for 12 s and was composed of 12 tones at an interval of 500–1000 ms between them. The total duration of the tone sequence for each trial was constant (i.e., 12 s). For the sequence of meter tones, the first tone (vs the second tone) was acoustically accented (+6 dB) to create the pattern of downbeats and upbeats, and this pattern was looped six times in each tone sequence. For the no-meter sequence, the tones were unaccented and had equal intensity (i.e., 40 dB above the threshold of individual sensation, collected before the experiment task). The tone sequences were varied across trials to avoid the practice effect, but were identical between the participants in a dyad.

The auditory stimuli were given to participants through two pairs of headphones (Philips) that were controlled by the same server with two 19-inch computer monitors. The monitors were placed on the middle of a table (110 × 80 cm) and were equipped with keyboards. Two participants of the dyad were seated at the table across from each other, each with her own computer monitor and keyboard. They each wore a pair of headphones to receive the auditory stimuli. They were also separated by a piece of white cardboard (110 × 80 cm) to block any visual information that might be used for communication.

#### Task and procedure

The participants of the dyads were asked to complete the finger-tapping task, that is, to tap their fingers to the auditory stimulus that they had just heard. Moreover, they were required to finger-tap along with their partner (the coordination task) after listening to the meter and the no-meter stimuli. During the coordination task, each participant received the auditory feedback of her partner’s response, and was asked to try her best to response synchronously with the partner. As a control, the participants were also instructed to tap their fingers with the computer (the independence task) after listening to the meter or no-meter stimuli. For the independence task, both participants received the auditory feedback of their own responses, and were asked to response synchronously with the auditory stimulus as precisely as possible.

The participants were first instructed on the experimental tasks and given several practice trials. They were then given a 20-s resting state, during which they were required to remain as motionless as possible and relax. The experimental tasks consisted of four blocks corresponding to the four conditions in this study (e.g., the participant heard the tap of her partner and was required to tap along with her), with a 30-s rest between blocks. The order of the four blocks was counterbalanced. Before each experimental task, one piece of instruction was presented on the display for 3 s to remind the participants of the task. Each block (i.e., performing the coordination/independence task with the meter/no-meter stimuli) consisted of 15 trials, and thus 60 trials were held in the entire experiment. For each trial, the participants first heard the meter/no-meter stimuli (i.e., 12 s), followed by a sound (262 Hz, 1000 ms) that served as a cue to remind them to start tapping their fingers. They were instructed to reproduce the stimuli that they had heard before by tapping their right index finger on the keyboard (participant #1: “f”; participant #2: “j”). During the experiment, the participants of each dyad were not allowed to communicate with each other through language or movement. We used the E-Prime software (Psychology Software Tools), which can provide the input and output of stimuli with millisecond-level precision. Specifically, the participants’ taps were collected through the response box (i.e., “f” and “j” on the keyboard device), and the feedback regarding the taps (i.e., the drip sound) were given to participants through SoundOut objects (that support audio file outputs) in E-prime.

#### Behavioral analysis

All trials were used in the behavioral and fNIRS analysis. To quantify behavioral coordination, we calculated the timing data of each participant’s taps during the coordination task in meter and no-meter conditions. We focused on the coordination task given our hypothesis (i.e., there is better behavioral coordination in the meter condition than in the no-meter condition). The onset time of the participants’ taps (i.e., the timestamp at which the participants began to tap) were extracted and then computed for the interpersonal time lag using the follow formula (Mu et al., 2016):

$$\delta_{inter{\_}_{i}}=\left|RT_{i,P1}-RT_{i,P2}\right|/(RT_{i,P1}+RT_{i,P2}),$$

where RT_i,P1_ and RT_i,P2_ are the times of the onset of taps by two participants of the same dyad at the i^th^ tap. The interpersonal time lag used here (instead of the raw values of lag, in seconds) was intended to eliminate the effects of differences in finger-tapping between members of a dyad as well as variance in the interval between tones presented in different trials from the behavioral index. The mean interpersonal time lag for taps was calculated to evaluate the overall behavioral coordination. A shorter lag indicated better behavioral coordination. To compare the measures of behavior between the meter and no-meter conditions, a linear mixed model was applied to the mean interpersonal time lag for the coordination task, including the fixed effect of the condition (meter vs no-meter) and individual differences (the mean interpersonal time lag in the independence task), and the random effect of the dyad. The significance of the model was assessed by using the likelihood ratio test.

To further estimate behavioral coordination, the trend and fluctuation of interpersonal time lags across taps were analyzed. Specifically, the interpersonal time lags were entered into detrended fluctuation analysis to estimate the Hurst exponent H (Kantelhardt et al., 2001). The value of H ranges from zero to one. If H is 0.5, the correlations are completely absent, and if H > 0.5, this implies a positive correlation. On the contrary, if H < 0.5, this implies a negative correlation. The interpersonal time lags were also transformed into the log scale because they followed a non-normal distribution, and were then regressed into the mixed linear regression model with taps and individual differences as the fixed factors, and the dyad as the random effect. The significance of the model was assessed using the likelihood ratio test.

#### fNIRS data recordings

**Figure 1.**
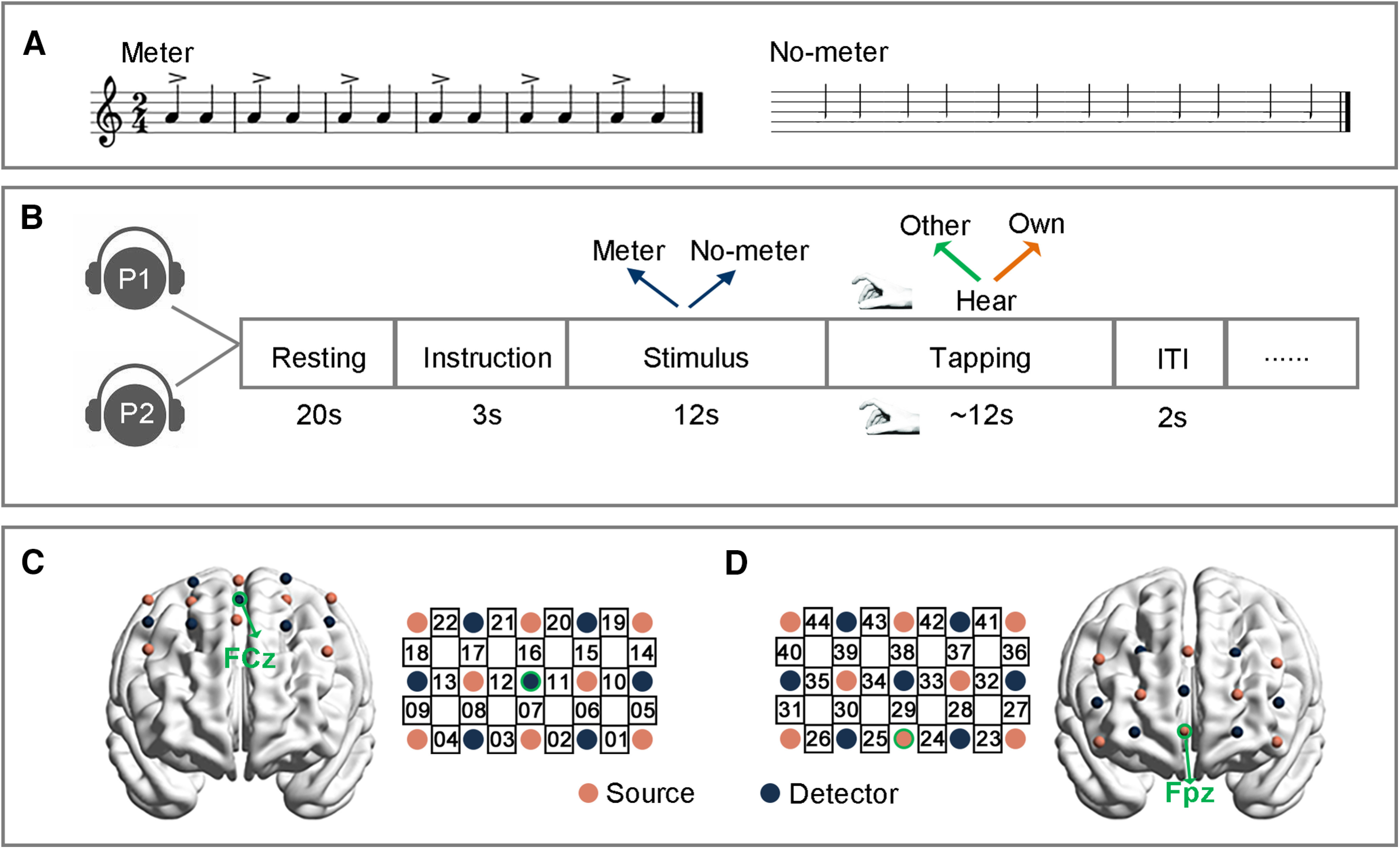
Experimental design. ***A***, Experimental stimulus. ***B***, Experimental procedure and task (P1, participant #1; P2, participant #2). ***C***, Probe configuration in experiment 1. ***D***, Probe configuration of a second patch in experiment 2.

fNIRS data were simultaneously recorded from both members of each dyad by using the ETG-7100 optical topography system (Hitachi Medical Corporation). The values of oxyHb and deoxyHb were obtained at a sampling rate of 10 Hz. One optode probe patch (3 × 5 setup) was placed on the head of each participant, and contained eight emitters and seven detectors that formed 22 measurement channels with a 3-cm separation between optodes. The middle optode of the second row of probes of the patch was placed at FCz (Fig. 1*B*), following the international 10–20 system (Okamoto et al., 2004). The correspondence between the channels of fNIRS and the measurement points on the cerebral cortex was determined by using the virtual registration method (Tsuzuki et al., 2007), which has been validated by a multisubject study on anatomic craniocerebral correlation. A 3D digitizer was used to measure the positions at which the channels existed on the head (Xiao et al., 2017), and the NIRS-SPM software for MATLAB was used to validate the standard brain model and data from the 3D digitizer (Ye et al., 2009). The possible MNI coordinates were thus obtained for each channel. Finally, the brain regions of the channels were determined based on the Automated Anatomical Labeling (AAL) atlas (Tzourio-Mazoyer et al., 2002) and checked through data from Neurosynth (a platform for large-scale and automated synthesis of fMRI data).

#### fNIRS data analysis

The raw data for each participant were preprocessed by using the correlation-based signal improvement method (Cui et al., 2010), which is based on the observation that the values of oxyHb and deoxyHb are usually negatively correlated but the motion of the head induces a positive correlation between them. The preprocessed signals were used to calculate the IBS for each dyad through the wavelet transform coherence (WTC) method, which can assess the cross-correlation between brain signals on the time–frequency plane (Grinsted et al., 2004).

To determine the frequency band of interest, the linear mixed model was used to compare IBS between experimental conditions across the full range of frequency (0.015–0.1 Hz). The frequencies above 0.1 Hz were removed as they might have been correlated with non-neuronal components, such as Mayer waves (around 0.1 Hz), respiration (0.2–0.6 Hz), and cardiac pulsation (above 0.7 Hz). The frequencies below 0.015 Hz were also removed because IBS has often been observed only at frequencies above 0.015 in fNIRS hyperscanning studies (Dai et al., 2018; Wang et al., 2019; Nguyen et al., 2021). A cluster-based permutation test was used, in the same manner as in a recent fNIRS hyperscanning study by us (Pan et al., 2022; Zhu et al., 2022). This method is a nonparametric statistical test that allows for multiple comparisons of multichannel (i.e., 22 channels) and multifrequency (i.e., 30 frequency bins) data, rather than familiar methods of correction (e.g., Bonferroni, FDR) based on a parametric statistical framework. It provides a straightforward way to solve the problem of multiple comparisons (Maris and Oostenveld, 2007). Nonparametric statistical tests offer complete freedom to use any test statistic one considers appropriate. This allowed us to solve the problem of multiple comparisons in a simple way.

The cluster-based permutation test consisted of six steps as follows. First, the IBS for each frequency and each channel was calculated by using the WTC for each experimental condition. Second, the IBS between meter and no-meter conditions was compared by using the frequency-by-frequency and channel-by-channel mixed linear model. Such model included the fixed effect of condition (meter vs no-meter) and individual differences (the IBS in the independence task), and the random effect of the dyad. The meter and no-meter conditions were compared directly (instead of relying on the task vs rest comparison) because of our hypothesis (i.e., there is enhanced IBS in interpersonal coordination in the presence of the meter relative to its absence). Moreover, it has been reported that an active task condition can offer a better baseline than the rest condition (Stark and Squire, 2001; Reindl et al., 2018). Third, the channels and frequencies that exhibited significant effect because of the condition (i.e., the meter condition > the no-meter condition, *p *< 0.05) were identified. Fourth, clusters with neighboring frequency bins (*N* ≥ 2) were formed. The statistics of each cluster were computed by averaging all the *F* values. Fifth, the above steps were repeated 1000 times by permuting the data. The permutation was constructed by randomly pairing the data for a participant from one dyad with that of another from a different dyad. Finally, the significant levels (*p *<* *0.05) were calculated by comparing the cluster statistic obtained from the dyads over 1000 permutations of randomized dyads.

### Results

#### Behavioral coordination

**Figure 2.**
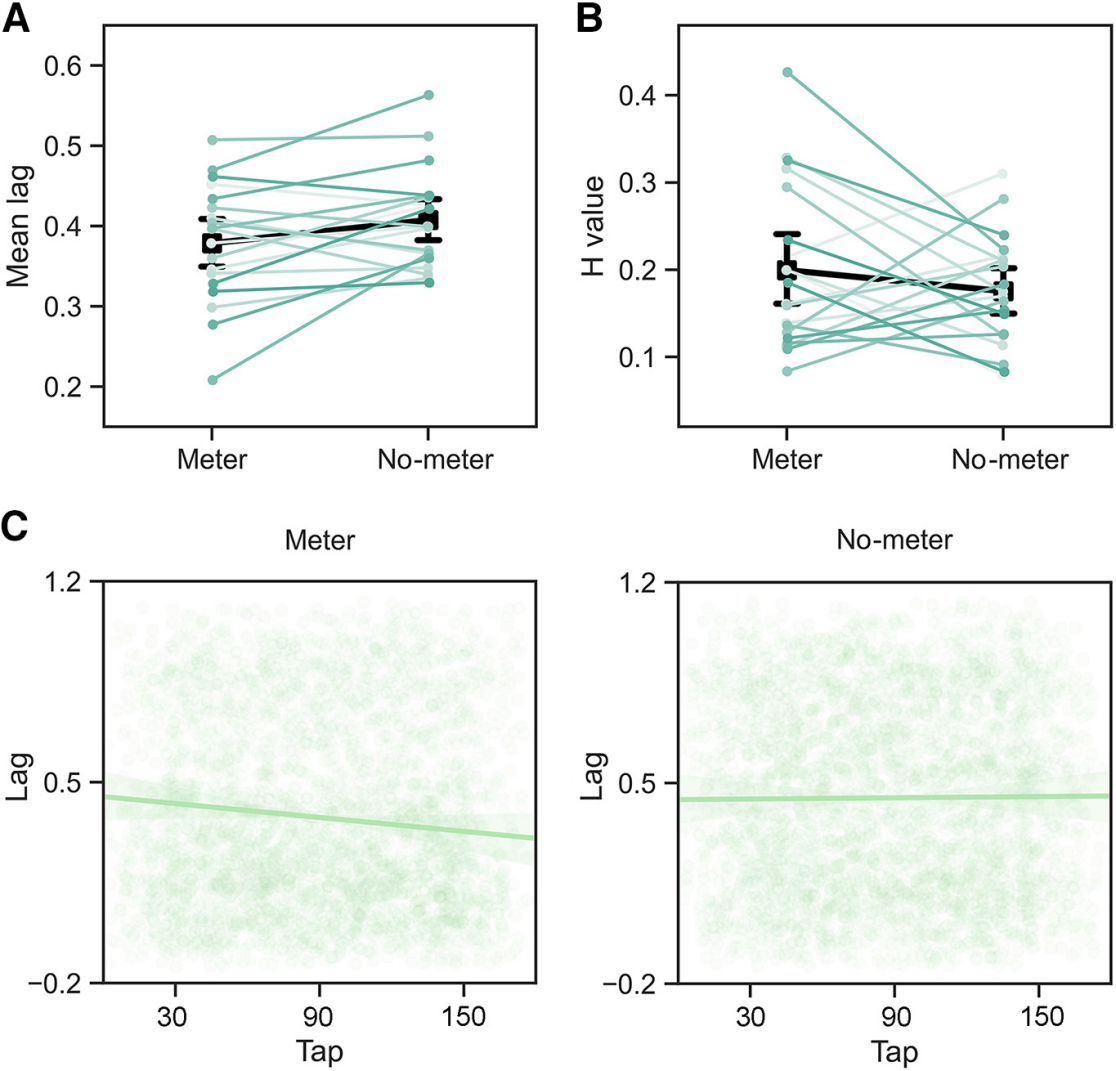
Behavioral performance on the coordination task between the meter and no-meter conditions in experiment 1. ***A***, Mean interpersonal time lag (Mean lag). Shorter mean interpersonal time lag in the meter condition compared with that in the no-meter condition. ***B***, Hurst exponent H of the detrended fluctuation analysis. There was no significant difference in H between the meter and the no-meter conditions. ***C***, The linear regression of interpersonal time lags across taps in the meter and no-meter conditions. The interpersonal time lags significantly decreased across taps in the meter condition. Colored lines indicate behavioral measures for each dyad. Black lines indicate the averaged values across dyads. Error bars represent SEMs.

The results of the linear mixed model showed that during the coordination task, the mean interpersonal time lag in the meter condition (mean ± deviation: 0.379 ± 0.071) was significantly shorter than in the no-meter condition [0.408 ± 0.060, *F *=* *5.086, *p *=* *0.037, *β* = −0.231, SE = 0.094, 95% confidence interval (CI) = −0.399 to −0.028; Fig. 2*A*]. These findings indicate better behavioral coordination of the dyads when exposed to the beat and the meter.

The detrended fluctuation analysis showed an insignificant difference between the meter (0.200 ± 0.091) and no-meter conditions (0.175 ± 0.062, *F *=* *1.294, *p *=* *0.270, *β* = 0.104, SE = 0.091, 95% CI = −0.076–0.282; Fig. 2*B*). Here, the values of H for each dyad were all <0.5, implying anticorrelations between interpersonal time lags across task trials, that is, a long interpersonal time lag would probably be followed by a short interpersonal time lag, and a short interpersonal time lag would probably be followed by a long interpersonal time lag.

In contrast, the results of linear regression analysis revealed that the interpersonal time lag significantly decreased across taps in the meter condition (*F *=* *4.168, *p *=* *0.041, *β* = −0.001, SE = 0.0003, 95% CI = −0.001 to −0.00003), and changed insignificantly in the no-meter condition (*F *=* *1.116, *p *=* *0.291, *β* = −0.0003, SE = 0.0003, 95% CI = −0.0003–0.001; Fig. 2*C*). These results indicate an improvement of coordination between interactors in the metrical context.

#### Enhanced IBS in interpersonal coordination with meter

**Figure 3.**
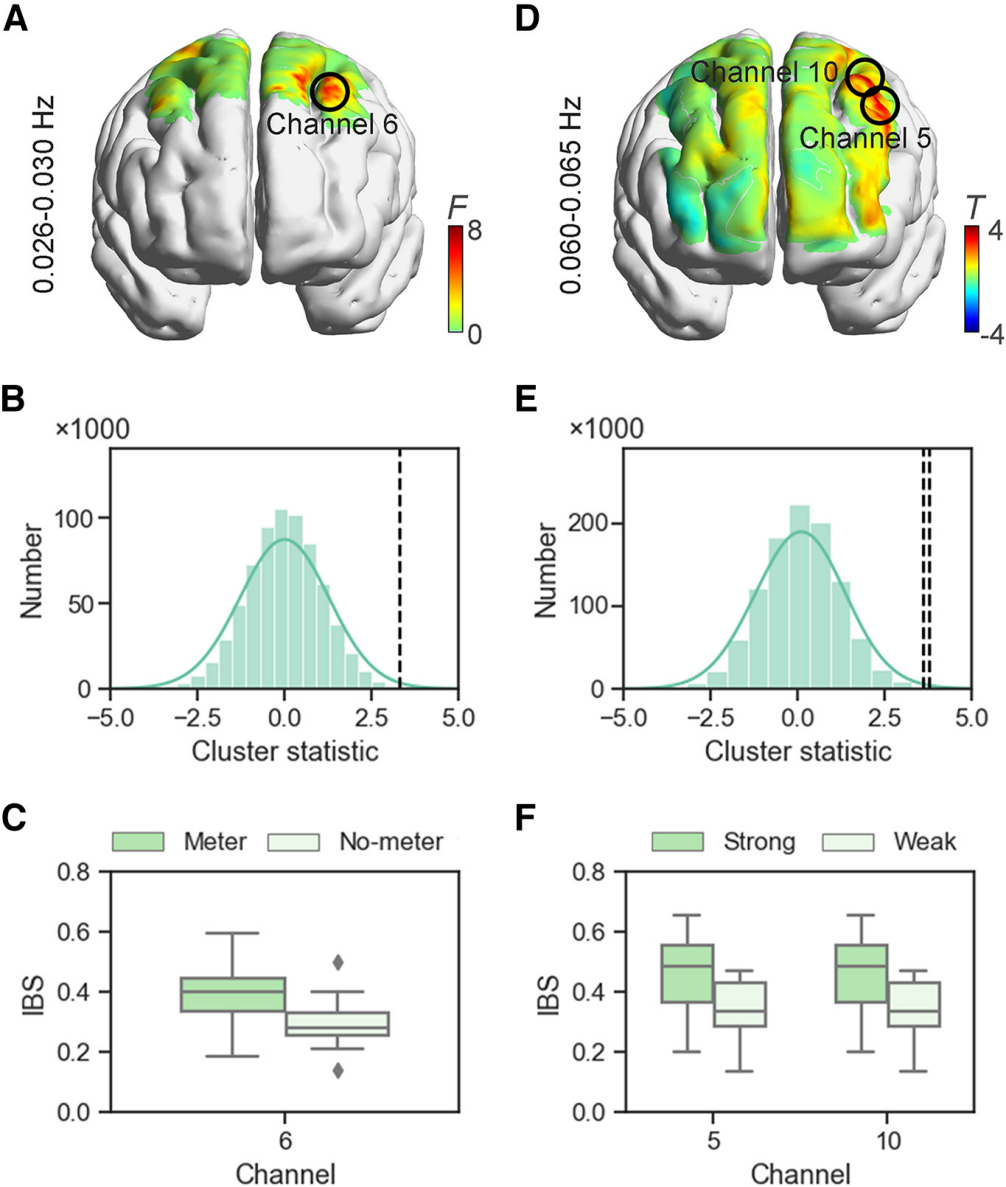
Interbrain synchronization (IBS) during the coordination task. The heat maps of the IBS in (***A***) experiment 1 and (***D***) experiment 2. Enhanced IBS was observed at channel 6 in the range of frequencies of 0.026–0.030 Hz in the meter condition compared with the no-meter condition. In experiment 2, the IBS in channels 5 and 10 (frequencies: 0.060–0.065 Hz) was greater in case of strong meters than weak meters. The distributions of the cluster statistic of the permutated data in (***B***) experiment 1 and (***E***) experiment 2. The black dashed lines indicate the positions of the cluster statistic of the pairs. The enhanced IBS in (***C***) experiment 1 and (***F***) experiment 2. Data were plotted through boxplots, in which the horizontal lines indicate median values, the boxes indicate the 25% and 75% quartiles, and the error bars represent the minimum/maximum values. The diamond dots represent the extreme values.

The cluster-based permutation test identified one channel–frequency cluster that reached significance: the cluster in channel 6 in the range of frequencies of 0.026–0.030 Hz. These frequency bands roughly corresponded to the duration of one task trial in experiment 1 (i.e., around 33 s based on the participants’ pragmatic response). In this range of frequencies (i.e., 0.026–0.030 Hz), the IBS in channel 6 was significantly greater in the meter condition (0.388 ± 0.111) than the no-meter condition (0.293 ± 0.075; Fig. 3*A*). According to the AAL atlas and data of Neurosynth, channel 6 was approximately located in the left-middle frontal cortex (MFC).

#### IBS is associated with behavioral coordination

**Figure 4.**
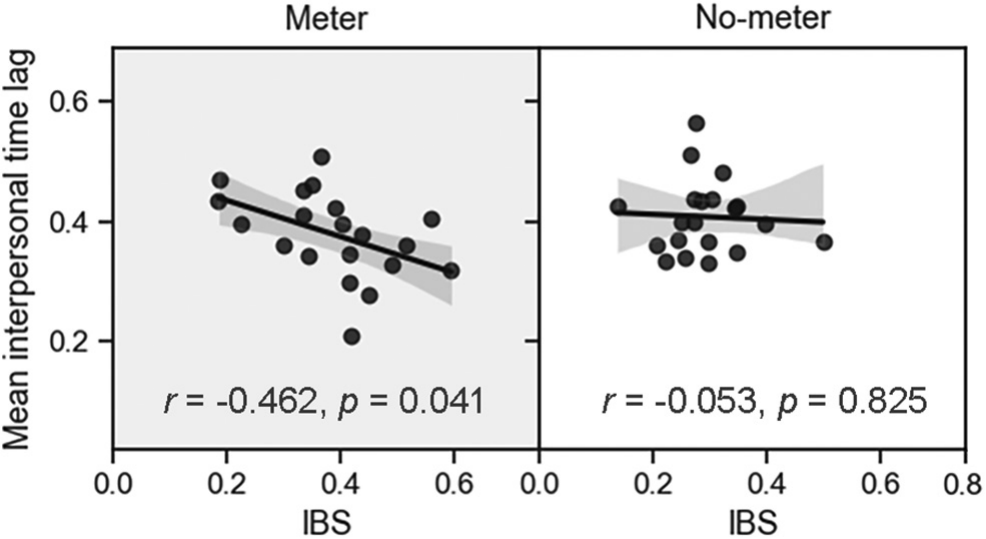
Correlation between interbrain synchronization (IBS) and behavioral coordination in experiment 1. The IBS at channel 6 during the coordination task was negatively associated with the mean interpersonal time lag in the meter condition. Each black point depicts a dyad of the mean interpersonal time lag of the participants (*y*-coordinate) and IBS at channel 6 (*x*-coordinate). The solid line represents the least-squares fit. The shaded area indicates the 95% confidence interval.

The correlation analysis revealed that the IBS at channel 6 in the coordination task was negatively correlated with the mean interpersonal time lag in the meter condition (*r* = −0.462, *p *=* *0.041; Fig. 4). This correlation was not significant in the no-meter condition (*r* = −0.053, *p *=* *0.825). These results indicate that the enhanced IBS at the middle frontal cortex was positively associated with behavioral coordination in case of exposure to the musical meter.

## Experiment 2

### Materials and Methods

The methods in experiment 2 were the same as in experiment 1, with the exceptions below.

#### Participants

A new group of 32 right-handed female students (mean age = 20.640 years, range: 18–28 years), comprising 16 random dyads, participated in experiment 2 in exchange for monetary compensation. The members of each dyad had not seen or known each other before.

#### Stimuli and apparatus

The stimuli were four kinds of meters generated by manipulating the accent (i.e., strong vs weak) and the frequency of occurrence (i.e., duple vs triple). For each tone sequence, there were 12 40-Hz pure tones at an interval of ∼500 ms between them. The total duration of the tone sequence for each trial was constant (i.e., 6 s). For the duple meters, every second tone was reduced in intensity by 10 or 2 dB, corresponding to strong and weak meters, respectively. For the triple meters, the intensities of every second and third tones were reduced by 10 dB (strong meters) or 2 dB (weak meters). These difference in loudness (i.e., 10 vs 2 dB) have been found in past work to induce significantly different responses (Ozertem and Erdogmus, 2008).

#### Task and procedure

Before the experimental task, the participants were asked to rest for 2 min to obtain more stable resting-state data from them (Lu et al., 2010). They completed the coordination task in the experiment, consisting of four blocks of eight trials yielded by crossing the accent (i.e., strong vs weak) and frequency of occurrence (i.e., duple vs triple). The order of the four blocks was counterbalanced.

#### Behavioral analysis

Only the mean interpersonal time lag was calculated to quantify the behavioral synchronization of the dyads in experiment 2, as insignificant values for the other behavioral measures (e.g., the value of H, the probability density) were recorded in the coordination task in experiment 1. Note that all trials were entered into the behavioral and fNIRS analysis.

#### NIRS data recordings

To obtain the brain signals of a more extended frontal cortex, two 3 × 5 patches were used in experiment 2. The first probe patch was set as in experiment 1, and generated 22 measurement channels (channels 1–22), while the second one covered the prefrontal area to form another 22 measurement channels (channels 23–44). The bottom row of the second patch was placed on top of the participant’ eyebrows, with the middle optrode at Fpz (Fig. 1*C*).

#### fNIRS data analysis

The same procedures (e.g., data preprocessing, the calculation of IBS, and the cluster-based permutation test) were used in experiment 2 as in experiment 1. But there was one exception: in the second step of the cluster-based permutation test, IBS between experimental conditions (i.e., strong vs weak) was compared by using the mixed linear model, in which only included the fixed effect of condition and the random effect of the dyad.

### Results

#### Behavioral coordination

**Figure 5.**
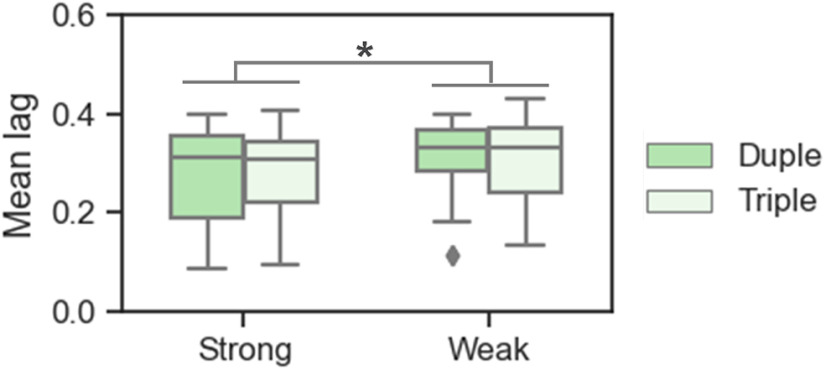
Behavioral coordination in experiment 2. A shorter mean interpersonal time lag (Mean lag) was observed in strong meters relative to weak meters. Boxplots are presented, with the horizontal lines indicating the median values, boxes indicating the 25% and 75% quartiles, and error bars representing the minimum/maximum values. The diamond dots represent the extreme values; *p <* *0.05.

To explore the effects of the frequency of occurrence and accent on behavioral coordination, the mixed linear regression model was used. The results showed a significant main effect of the accent (*F *=* *6.954, *p *=* *0.011, *β* = −0.199, SE = 0.076, 95% CI = −0.347 to −0.051), with a shorter mean interpersonal time lag in case of strong meters (0.273 ± 0.101) than weak meters (0.305 ± 0.088; Fig. 5). The main effect of the frequency of occurrence, and the interaction between the frequency of occurrence and the accent were insignificant (*F*s* *<* *0.370, *p*s* *>* *0.546). To clarify how accent affected behavioral coordination, the interpersonal time lags of the accented tones were extracted and compared between the strong (i.e., +10 dB) and weak (+2 dB) tones by using the mixed linear regression model. The results revealed significantly shorter interpersonal time lags for strong tones (0.280 ± 0.093) than for weak tones (0.306 ± 0.082; *F *=* *4.851, *p *=* *0.044, *β* = −0.149, SE = 0.068, 95% CI = −0.286 to −0.013). These results suggest that accents promoted behavioral coordination during the coordination task in metrical contexts, with better behavioral synchronization in case of strong meters and tones.

#### Increased IBS because of accent

**Figure 6.**
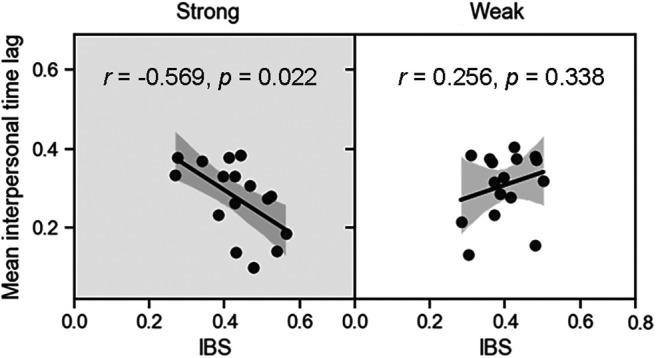
Correlation between interbrain synchronization (IBS) and behavioral coordination in experiment 2. The average IBS at channels 5 and 10 was negatively associated with the mean interpersonal time lag in case of strong meters. The values in duple and triple meters were averaged. Each black point depicts a dyad of the mean interpersonal time lag of participants (*y*-coordinate) and the average IBS in channels 5 and 10 (*x*-coordinate). The solid line represents the least-squares fit. The shaded area indicates the 95% confidence interval.

The IBS was compared between the conditions (strong vs weak) by using the paired *t* test because we observed a significant effect of the accent on behavioral coordination. The cluster-based permutation test revealed two significant channel–frequency clusters: cluster 1 (frequencies 0.060–0.065 Hz, channel 5) and cluster 2 (frequencies 0.060–0.065 Hz, channel 10). The significant frequency bands were also in line with the duration of a task trial (i.e., around 15 s in experiment 2). For the significant frequencies (i.e., 0.060–0.065 Hz), the IBS at channels 5 and 10 was higher in case of a strong meter (channel 5: 0.448 ± 0.132; channel 10: 0.451 ± 0.082) than a weak meter (channel 5: 0.341 ± 0.092; channel 10: 0.345 ± 0.105; Fig. 3*F*). Both channels 5 and 10 were roughly located at the left-middle frontal cortex according to the results of fNIRS localization. Because these two channels belonged to the same region of the brain, they were averaged into one IBS. This IBS was negatively correlated with the mean interpersonal time lag in case of a strong meter (*r* = −0.569, *p *=* *0.022; Fig. 6). However, the corresponding correlation was not significant in case of a weak meter (*r *=* *0.256, *p *=* *0.338).

## Discussion

This study investigated the functional role of IBS, in the presence of musical meter, on interpersonal coordination through two fNIRS hyperscanning experiments. Experiment 1 revealed better behavioral coordination when the subjects were exposed to musical meter, as indexed by shorter mean interpersonal time lags of taps in the meter condition than the no-meter condition. Over time, the coordination of dyads improved when they were exposed to musical meter. These findings suggest that people dynamically adapt with others during interpersonal coordination. When musical meter is involved, their mutual adaption begins to be more effective and this leads to good behavioral coordination. Experiment 1 also revealed greater IBS at the left middle frontal cortex (MFC) when the dyads coordinated in a metrical context than in a no-metrical context. The MFC-IBS was positively correlated with behavioral coordination in the metrical context. Our fNIRS results are consistent with previous findings, in which the MFC-IBS was observed during scenarios involving social interaction, including decision-making (Zhang et al., 2017; Xie et al., 2022), cooperation (Hu et al., 2017; Feng et al., 2020), and communication (Balconi et al., 2021). In particular in various cooperative activities, pairs of participants showed enhanced MFC-IBS in case of specific populations and tasks, such as professional athletes (Li et al., 2020), expert teachers (Sun et al., 2020), and less creative individuals (Xue et al., 2018), and providing positive feedback (Lu et al., 2019a) and demanding divergent thinking (Lu et al., 2019b). Moreover, such enhanced MFC-IBS has been reported to be correlated with behavioral cooperation (Lu and Hao, 2019).

Why does music increase our coordination with others? Several theoretical frameworks have been proposed to explain this phenomenon. One possible explanation is that music and interpersonal coordination share neural resources (e.g., the premotor and supplementary motor areas, which belong to the mirror system) at the level of predicting stimuli (Novembre et al., 2012) and information integration (Loehr et al., 2013). Exposure to music can thus facilitate the general processing in case of interpersonal coordination (Keller et al., 2014). Accordingly, the IBS should be located in the area of the mirror system, but this is not supported by the findings of this study. Another explanation emphasizes that music regulates interpersonal coordination through endogenic rhythm (Phillips-Silver et al., 2010). Musical beat and meter are like the natural rhythm of our bodies, like breathing and heartbeat, and relate naturally to motion. A direct causal link has been reported between endogenous rhythms and interpersonal synchrony in a music performance task (Zamm et al., 2016). However, the IBS in our study might be uncorrelated with the endogenic rhythm (e.g., breathing and heartbeats) because of the auditory stimuli used here. Our fNIRS findings can be understood within the view that music functions as a kind of a “social glue” that brings people together (Demos et al., 2012). The MFC-IBS has been suggested to be related to our understanding of others’ mental states and behaviors. For example, the perspective-taking scores of the participants were positively correlated with the MFC-IBS during cooperation (Sun et al., 2020). Another study found a negative correlation between the MFC-IBS and the participants’ agreeableness-related trait when they made defection decisions (Zhang et al., 2021). Thus, the increased MFC-IBS here may be related to the process whereby participants pay more attention to their partners’ actions, and make a greater effort to understand and coordinate with them in musical contexts. Our fNIRS results provide a fundamental neural mechanism for the promotional effect of music on interpersonal coordination.

Notably, experiment 2 showed that the MFC-IBS and behavioral coordination were enhanced by strong meters relative to weak meters. These results are in line with previous findings, where greater active brain activity and accurate behavioral performance were observed when processing strong meters over weak meters (Patel et al., 2005; Kung et al., 2013). These results can be explained by signal detection theory, in which the intensity of the stimulus plays an important role in information processing (Green and Swets, 1966). In case of competition between sensory signals for limited channel capacity, the intensity of each stimulus becomes paramount and the accented stimulus is a priority (Macmillan and Creelman, 2004). Indeed, strong meters are known to more easily attract the attention of listeners relative to weak meters (Kantardjieff and Jones, 1997). Compared with soft music, music with accented beats and meters can facilitate greater dissociation from the internal sensations of fatigue (Hutchinson and Sherman, 2014). Thus, our results extend research on the benefits of accented stimuli for brain-to-brain coupling during interpersonal coordination in the presence of musical meter.

With regard to the frequency of occurrence of meters, the results in experiment 2 indicated an insignificant difference in IBS and behavioral coordination between duple and triple meters. This appears to be inconsistent with previous studies, in which the frequency of occurrence of meters was found to influence human cognition and behavior (Grahn and Brett, 2007; Grahn and Rowe, 2009). For instance, duple meters are easier to coordinate as they are “more natural” than triple meters as the former are similar to the endogenous rhythms of humans (i.e., pulse, heartbeat). Previous EEG studies have reported that compared with duple meters, triple meters awakened a larger amplitude of P300 (Jongsma et al., 2004) that was associated with a wider range of sensorimotor and frontoparietal areas (Fujioka et al., 2015). This suggests that triple meters might require more processing capacities. Although it is difficult to interpret a null finding, it should be noted that the bias between duple and triple meters can be learned, and is not universal across individuals (Hannon and Trainor, 2007; Trainor and Hannon, 2013). Future studies should examine the brain-to-brain coupling in the presence of duple and triple meters during interpersonal coordination with music while controlling for prior knowledge and individual differences.

In this study, we used a beat reproduction task in which the participants were asked to reproduce a sequence of the stimuli after having heard it (Grahn and Brett, 2007). This reproduction task is different from the beat tapping task (e.g., a finger-tapping task in synchrony with a given beat) because the former task also requires a memory component (Steinbrink et al., 2019). The significant IBS reported here might have thus been obtained because of the difference between the memory required for metered/accented tones and that for no-metered tones. However, a significant MFC-IBS was not observed when comparing the meter and no-meter conditions on the independence task by using the paired *t* test. In addition, a previous fMRI study showed that the temporal lobe was correlated with the capacity of the auditory working memory during a rhythm reproduction task (Grahn and Schuit, 2012). Thus, the enhanced IBS reported in this study seems to be independent of the memory component. To confirm our findings, future work should explore the significant IBS reported here through the beat tapping task.

Several limitations of this work should be addressed in future research. First, the experiments reported here involved only female participants. As mentioned before, musical meters are not perceived in the same way by all populations, and a range of interindividual differences are generally obtained (Schaefer and Overy, 2015). There is also evidence that the IBS during interpersonal coordination differs in males and females, with greater IBS in female-female pairs than male-male pairs (Mu et al., 2016), and stronger IBS between male-female pairs than same gender pairs (i.e., male-female pairs, female-female pairs; Cheng et al., 2015). It would be interesting to explore the IBS in dyads with partners with opposite gender (i.e., male-female pairs) during interpersonal coordination with meters in the future study. Second, the stimuli of meters used here were simple, and did not contain temporal information (i.e., pitch, volume, and timbre) as such meters can move participants to internally feel the meter (Grahn and Brett, 2007). In everyday life, however, we also experience compound meters that have time signatures indicating the number of beats as a multiple of three. Previous neuroimaging studies have shown that simple and compound meters elicit different levels of brain activity. For example, greater activity in the putamen was identified for simple meters than compound meters and the absence of meter (Teki et al., 2011). Future work should examine the IBS during interpersonal coordination with compound meters. Finally, the IBS reported here was found to be approximately located at the MFC in different channels in our two fNIRS hyperscanning experiments (i.e., channel 6 in experiment 1, and channels 5 and 10 in experiment 2). Although fNIRS is an ideal choice for neuroscience research in a natural environment, it has a relatively poor spatial resolution (∼1–3 cm; Boas et al., 2004). Future work should investigate brain-to-brain coupling during interpersonal metrical coordination by using other neural technologies with a high spatial resolution, such as fMRI hyperscanning.

In conclusion, this study provided direct evidence for enhanced IBS during interpersonal coordination in the presence of the musical meter, where this provides a potential neural marker of interpersonal metrical coordination. We have argued that the enhanced IBS at the middle frontal cortex reflects greater attention to and understanding of the partner’s actions. This extends our knowledge of the brain mechanism underlying the effects of music on interpersonal coordination.

## Synthesis

Reviewing Editor: Christine Portfors, Washington State University Decisions are customarily a result of the Reviewing Editor and the peer reviewers coming together and discussing their recommendations until a consensus is reached. When revisions are invited, a fact-based synthesis statement explaining their decision and outlining what is needed to prepare a revision will be listed below. The following reviewer(s) agreed to reveal their identity: Alexander Stevens.

As the paper stands now, the results of the studies are too vague to provide a substantive advance in our understanding of the role of music in interpersonal coordination. However, if the authors were to include the additional data that they collected on the independent task, and use that data in a regression model to account for individual differences, they would be able to draw stronger conclusions about the sources driving coordination in the different task conditions.

Reviewer comments:

The main goal of this manuscript is to identify brain correlates of inter-personal coordination arising from music. The authors pose the hypothesis that there is better behavioral coordination (tapping) during metered versus non-metered tones (exp 1), or during strongly versus weakly accented tones (exp 2). They used fNIRS to record frontal cortical hemodynamic changes during the tasks to search for physiological correlates of dyadic synchrony. The main findings indicate that pairs of individuals show greater synchrony in tapping to the metered compared to non-metered (exp 1) tone strings, and accented compared to unaccented (exp 2) tone strings, in support of the hypothesis.

While the study is generally well constructed, there are some shortcomings that preclude drawing clear conclusions from the data as they are currently presented. Most of these issues are addressable by including data from the Independence task that the investigators collected but did not report. I explain this below.

A major point of confusion for me is that it sounds as if participants were given potentially competing instructions for the coordination task. In describing the task instructions, the manuscript states (line 151,152), “Participants were asked to...tapping their fingers to the auditory stimulus they just heard”. Yet on line 155-156), they were “also instructed to try their best to response [sic] synchronously with the partner”. Then (line 173), “they were instructed to reproduce the stimulus they heard before by tapping their right index finger on the keyboard”. While the researchers contend that the improvement in interpersonal lag across trials in the metered condition reflects the “dyads continuously adapted their behavior” (line 311), this may reflect the conflicting instructions. How the participants interpret these instructions could lead to greater variability until the dyad settles on a strategy. It could be that one member of the dyad maintains a rhythm and the second member adjusts to that member, an effect reported by Konvalinka et al. (2014). It is also possible that within dyads, individuals relied on the tapping pattern of their partner and tried to converge, without attempting to maintain the sample stimulus. The point is, it may have nothing to do with the metered condition.

It could be possible to disentangle the influences on coordination under the different task conditions by including the data from the Independent task. This would provide a clean measure of each participant’s ability to reproduce the stimulus pattern with no competing stimuli from a dyadic partner. This has two advantages: 1) it would provide a measure of individual differences in pattern reproduction, which is critical to dyad performance. 2) it would provide evidence for the effects of metered v non-metered stimuli on individual memory and reproduction of the tone stimuli. The contribution of individual lags in the independent condition (metered and unmetered) could be used to estimate sources of variability in dyad lags in the coordination conditions. This approach would provide a more nuanced understanding of how coordination arises, and what elements of individual differences may contribute to better synchronization of dyads in the metered compared to non-metered conditions.

The lack of a link between the temporal components of the task trials and the significant frequency bands (Exp 1: 0.026-0.03 Hz) in the fNIRS data is concerning. This frequency band was found in Experimebnt 2 (although a few others bands were). Why would these bands vary across the two experiments if they are supposedly reflecting similar mechanisms of IBS? The concern here is that mass univariate analyses of hemodynamic data are highly prone to spurious correlations in small samples (Marek et al., 2022). Here again, the fNIRS data from the unreported Independent Task conditions could similarly provide further validation for the channels and frequency bands identified as relevant to interpersonal lag in the coordination task. It seems that the stimulus recall in the Independent task should be similar (and easier) than the synchronization required in the coordination task, but would still rely on the same brain systems. Convergent data from the Independent task would strengthen the confidence that the fNIRS data is capturing task relevant information.

As raised in the previous reviews, the trial structure is still unclear:

Line 133 “Each tone sequence lasted for 12 seconds longer, which was composed of 12 tones with an interval of 500-1000 ms between each tone”. Were the tone sequences always 12 seconds? If so, then with shorter inter-stimulus intervals, there would need to be more tones. Conversely, if each trials consisted of 12 tones, then the trial length will vary as a function of the inter-stimulus interval. This should be clarified.

Lines 209-212: “We summed...”. This seems to suggest that only trials with interpersonal time lags less than the median were retained. Perhaps I am misunderstanding this but it seems to simply state the smaller interpersonal gaps indicated better synchronization. If this is the data used in subsequent analyses, it could strongly bias the analyses in favor of the hypothesized effect of better metered v. non-metered coordination. It would be helpful to clarify the purpose of this measure and what it is telling us about the data (results ported on lines 297-300).

Despite reasonably good statistical reporting, there are several areas where more details are needed. In particular, the method of correction for multiple comparisons of the individual subject fNIRS data paired t-tests between the metered and non-metered conditions (channels x frequency bands) suggests that there is a correction for multiple comparisons, but what correction and how it is applied is not described (e.g., lines 273-274). If there is no correction applied, there needs to be.

These comments apply as well to Experiment 2.

Other comments:

Line 288-289: the reported statistics are not identified (mean {plus minus} sem?)

The purpose of Figure 2B is unclear. It seems wholly descriptive and the meaning of the boxed region is poorly explained.

Although the mean lags are smaller for metered than non-metered stimuli, the variance (or at least the range) for the metered conditions is larger than the non-metered condition (line 288 and Fig 2a). This is counter-intuitive and suggests there is greater variability among the dyads in the metered condition.

The design of the experiments really tests whether the memory for metered and accented tones are more effective in synchronizing behavior than memory for non-metered tones, because as I understand it, the subject repeats a sequence after hearing it. This distinction between a remembered and a present rhythm is important because in the current case it is not the “music” per se that is driving the coordination between individuals, but the ability of the individuals to accurately retain the rhythm.

The error bars and shaded regions of the bar charts need to be defined.

While the writing is generally good, there are some grammatical and syntactic errors that make the manuscript difficult to understand, and it would benefit from editing by a fluent English speaker.

## Author Response

RESPONSES TO EDITOR AND REVIEWERS (eN-NWR-0504-21R1)

We really appreciate the comments and suggestions from both editor and reviewers regarding our article submission entitled “Musical meter induces inter-personal brain synchronization during their coordination” (eN-NWR-0504-21R1). In the revised manuscript, we have tried our best to address all comments and questions with corrections in blue color. Below we have made a point-by-point reply to each comment offered by the reviewers.

Synthesis Statement for Author (Required):

As the paper stands now, the results of the studies are too vague to provide a substantive advance in our understanding of the role of music in interpersonal coordination. However, if the authors were to include the additional data that they collected on the independent task, and use that data in a regression model to account for individual differences, they would be able to draw stronger conclusions about the sources driving coordination in the different task conditions.

Response: Done as suggested. We have conducted the regression model with the data of the independence task. Specifically, in Experiment 1, the linear mixed model was conducted on the behavioral measures of the coordination task, including a fixed effect of the condition (i.e., meter vs. no-meter), a fixed effect of individual differences (i.e., the measures of the independence task), and a random effect of the dyad. The reanalysis of the IBS in Experiment 1 was the same as that of the behavioral measures. The new results were similar with those in the original paper.

In the revised paper, we have added the related information and rewritten the words in Methods (line 201-206 in page 9, line 225-227 in page 10, line 236-239 in page 10, line 289-291 in page 12) as follows:

"Behavioral analysis. To quantify behavioral coordination, we calculated the timing data of each participant’s taps during the coordination task in meter and no-meter conditions. We focused on the coordination task given our hypothesis (i.e., there is better behavioral coordination in the meter condition than in the no-meter condition). The onset time of the participants’ taps (i.e., the timestamp at which the participants began to tap) were extracted and then computed for the inter-personal time lag using the follow formula (Mu et al., 2016):

δ_(inte〖r_〗_i)=|(〖RT〗_(i,P1)-〖RT〗_(i,P2))|/(〖RT〗_(i,P1)+〖RT〗_(i,P2))

where RTi,P1 and RTi,P2 are the times of the onset of taps by two participants of the same dyad at the ith tap. The inter-personal time lag used here (instead of the raw values of lag, in seconds) was intended to eliminate the effects of differences in finger-tapping between members of a dyad as well as variance in the interval between tones presented in different trials from the behavioral index. The mean inter-personal time lag for taps was calculated to evaluate the overall behavioral coordination. A shorter lag indicated better behavioral coordination. To compare the measures of behavior between the meter and no-meter conditions, a linear mixed model was applied to the mean inter-personal time lag for the coordination task, including the fixed effect of the condition (meter vs. no-meter) and individual differences (the mean inter-personal time lag on the independence task), and the random effect of the dyad. The significance of the model was assessed by using the likelihood ratio test.”

"The estimated probability densities with short inter-personal time lags (i.e., shorter than the median; 0.4 for our dataset) were summed and compared between the experimental conditions. All trials were entered into the kernel density estimation. However, only probability densities with short inter-personal time lags were retained in the linear mixed model, with the fixed effects of the condition and individual differences as well as the random effect of the dyad. A higher probability density of taps with short inter-personal time lags for dyads also manifested better behavioral coordination.

To estimate behavioral coordination, the trend and fluctuation of inter-personal time lags across taps were analyzed. Specifically, the inter-personal time lags were entered into detrended fluctuation analysis to estimate the Hurst exponent H (Kantelhardt et al., 2001). The value of H ranges from zero to one. If H is 0.5, the correlations are completely absent, and if H > 0.5, this implies a positive correlation. On the contrary, if H < 0.5, this implies a negative correlation. The inter-personal time lags were also transformed into the log scale because they followed a non-normal distribution, and were then regressed into the mixed linear regression model with taps and individual differences as the fixed factors, and the dyad as the random effect. The significance of the model was assessed using the likelihood ratio test.”

"The cluster-based permutation test consisted of six steps as follows: First, the IBS for each frequency and each channel was calculated by using the WTC for each experimental condition. Second, the IBS between meter and no-meter conditions was compared by using the frequency-by-frequency and channel-by-channel mixed linear model. The meter and no-meter conditions were compared directly (instead of relying on the task vs. rest comparison) due to our hypothesis (i.e., there is enhanced IBS in inter-personal coordination in the presence of the meter relative to its absence). Moreover, it has been reported that an active task condition can offer a better baseline than the rest condition (Reindl et al., 2018; Stark & Squire, 2001). Third, the channels and frequencies that exhibited significant effect due to the condition (i.e., the meter condition > the no-meter condition, p < 0.05) were identified. Fourth, clusters with neighboring frequency bins (N {greater than or equal to} 2) were formed. The statistics of each cluster were computed by averaging all the F values. Fifth, the above steps were repeated 1,000 times by permuting the data. The permutation was constructed by randomly pairing the data for a participant from one dyad with that of another from a different dyad. Finally, the significant levels (p < 0.05) were calculated by comparing the cluster statistic obtained from the dyads over 1,000 permutations of randomized dyads.”

Second, we have we updated the new results in Results in the revision (line 306-348 in page 13-15) as follows:

"2.2 Results

Behavioral coordination. The results of the linear mixed model showed that during the coordination task, the mean inter-personal time lag in the meter condition (mean {plus minus} deviation: 0.379 {plus minus} 0.071) was significantly shorter than in the no-meter condition (0.408 {plus minus} 0.060, F = 5.086, p = 0.037, β = -0.231, SE = 0.094, 95% confidence interval (CI) = -0.399 to -0.028; Figure 2A). These findings indicated better behavioral coordination of the dyads when exposure to the beat and the meter. In line with this, the results of linear regression analysis revealed that the inter-personal time lag significantly decreased across taps in the meter condition (F = 4.168, p = 0.041, β = -0.001, SE = 0.0003, 95% CI = -0.001 to -0.00003), and changed insignificantly in the no-meter condition (F = 1.116, p = 0.291, β = -0.0003, SE = 0.0003, 95% CI = -0.0003 to 0.001; Figure 2D). These results suggest effective mutual adaptation between interactors in the metrical context.

By contrast, the probability densities of short inter-personal time lags (i.e., 0 < inter-personal time lag < 0.4) were not significantly different between the meter condition (18.242 {plus minus} 2.753) and the no-meter condition (17.723 {plus minus} 2.837, F = 1.210, p = 0.285, β = 0.163, SE = 0.148, 95% CI = -0.132 to 0.459; Figure 2B). Detrended fluctuation analysis also showed an insignificant difference between the meter (0.200 {plus minus} 0.091) and no-meter conditions (0.175 {plus minus} 0.062, F = 1.294, p = 0.270, β = 0.104, SE = 0.091, 95% CI = -0.076 to 0.282; Figure 2C). The value of H in the coordination task was less than 0.5, implying negative long-range correlations between inter-personal time lags, that is, a long inter-personal time lag would probably be followed be a short inter-personal time lag, and vice versa. These findings indicated weaker negative long-range correlations, suggesting a more volatile trend, between inter-personal time lags in the coordination task. This in turn suggested continuous mutual adaptation between the partners during inter-personal coordination.

Taken together, these results indicate that regardless of whether they were in a metrical context, the dyads continuously adapted their behaviors with respect to each other and showed superior behavioral performance during inter-personal coordination. Moreover, when exposed to the meter, their continuous mutual adaptation became effective and they showed better behavioral coordination during inter-personal coordination.

Enhanced IBS in inter-personal coordination with meter. The cluster-based permutation test identified one channel-frequency cluster that reached significance: the cluster in channel 6 in the range of frequencies of 0.026-0.030 Hz. These frequency bands roughly corresponded to the duration of one task trial in Experiment 1 (i.e., around 33 s based on the participants’ pragmatic response). In this range of frequencies (i.e., 0.026-0.030 Hz), the IBS in channel 6 was significantly greater in the meter condition (0.388 {plus minus} 0.111) than the no-meter condition (0.293 {plus minus} 0.075; Figure 3A). According to the AAL atlas and data of Neurosynth, channel 6 was approximately located in the left-middle frontal cortex.”

Last, we have also updated Figure 3 with the new statistics when analyzing our data by using the linear mixed model. In the revision, Figure 3 has been showed as follows:

"Figure 3. Inter-brain synchronization (IBS) during the coordination task. The heat maps of the IBS in (A) Experiment 1 and (D) Experiment 2. Enhanced IBS was observed at channel 6 in the range of frequencies of 0.026-0.030 Hz in the meter condition compared with the no-meter condition. In Experiment 2, the IBS in channels 5 and 10 (frequencies: 0.060-0.065 Hz) was greater in case of strong meters than weak meters. The distributions of the cluster statistic of the permutated data in (B) Experiment 1 and (E) Experiment 2. The black dashed lines indicate the positions of the cluster statistic of the pairs. The enhanced IBS in (C) Experiment 1 and (F) Experiment 2. Data were plotted through boxplots, in which the horizontal lines indicate median values, the boxes indicate the 25% and 75% quartiles, and the error bars represent the minimum/maximum values. The diamond dots represent the extreme values.”

The main goal of this manuscript is to identify brain correlates of inter-personal coordination arising from music. The authors pose the hypothesis that there is better behavioral coordination (tapping) during metered versus non-metered tones (exp 1), or during strongly versus weakly accented tones (exp 2). They used fNIRS to record frontal cortical hemodynamic changes during the tasks to search for physiological correlates of dyadic synchrony. The main findings indicate that pairs of individuals show greater synchrony in tapping to the metered compared to non-metered (exp 1) tone strings, and accented compared to unaccented (exp 2) tone strings, in support of the hypothesis.

While the study is generally well constructed, there are some shortcomings that preclude drawing clear conclusions from the data as they are currently presented. Most of these issues are addressable by including data from the Independence task that the investigators collected but did not report. I explain this below.

A major point of confusion for me is that it sounds as if participants were given potentially competing instructions for the coordination task. In describing the task instructions, the manuscript states (line 151,152), “Participants were asked to...tapping their fingers to the auditory stimulus they just heard”. Yet on line 155-156), they were “also instructed to try their best to response [sic] synchronously with the partner”. Then (line 173), “they were instructed to reproduce the stimulus they heard before by tapping their right index finger on the keyboard”. While the researchers contend that the improvement in interpersonal lag across trials in the metered condition reflects the “dyads continuously adapted their behavior” (line 311), this may reflect the conflicting instructions. How the participants interpret these instructions could lead to greater variability until the dyad settles on a strategy. It could be that one member of the dyad maintains a rhythm and the second member adjusts to that member, an effect reported by Konvalinka et al. (2014). It is also possible that within dyads, individuals relied on the tapping pattern of their partner and tried to converge, without attempting to maintain the sample stimulus. The point is, it may have nothing to do with the metered condition.

It could be possible to disentangle the influences on coordination under the different task conditions by including the data from the Independent task. This would provide a clean measure of each participant’s ability to reproduce the stimulus pattern with no competing stimuli from a dyadic partner. This has two advantages: 1) it would provide a measure of individual differences in pattern reproduction, which is critical to dyad performance. 2) it would provide evidence for the effects of metered v non-metered stimuli on individual memory and reproduction of the tone stimuli. The contribution of individual lags in the independent condition (metered and unmetered) could be used to estimate sources of variability in dyad lags in the coordination conditions. This approach would provide a more nuanced understanding of how coordination arises, and what elements of individual differences may contribute to better synchronization of dyads in the metered compared to non-metered conditions.

Response: Yes, two instructions were given to the participants: to keep the given beat as precisely as possible, while at the same time synchronizing with the partner/computer (i.e., the tap sound they heard when they were tapping). To note, the tap sound of the computer was the tap of the participants themselves.

We agree that it is possible that participants only coordinate with their partner without concerning the meter/no-meter. The two instructions might be competing. As you suggested, we have tried to analysis the data in the independence task. Specially, we have performed the linear mixed model with a fixed effect of the condition (i.e., meter vs. no-meter), a fixed effect of individual differences (i.e., the data of the independence task), and a random effect of the dyad. Then we found similar results that reported in the original paper (Please see more details in the previous question). In this way, the revealed effects in our study cannot be interpreted in terms of individual differences (e.g., the interactive pattern of one dyad, temporal reproduction, and working memory).

The lack of a link between the temporal components of the task trials and the significant frequency bands (Exp 1: 0.026-0.03 Hz) in the fNIRS data is concerning. This frequency band was found in Experiment 2 (although a few others bands were). Why would these bands vary across the two experiments if they are supposedly reflecting similar mechanisms of IBS? The concern here is that mass univariate analyses of hemodynamic data are highly prone to spurious correlations in small samples (Marek et al., 2022). Here again, the fNIRS data from the unreported Independent Task conditions could similarly provide further validation for the channels and frequency bands identified as relevant to interpersonal lag in the coordination task. It seems that the stimulus recall in the Independent task should be similar (and easier) than the synchronization required in the coordination task, but would still rely on the same brain systems. Convergent data from the Independent task would strengthen the confidence that the fNIRS data is capturing task relevant information.

Response: Yes, the significant frequency bands were different in our two experiments. In Experiment 1, the significant frequency bands ranged from 0.026 to 0.030 Hz, which roughly corresponded with the duration of one task trial (i.e., around 33 seconds based on participants’ pragmatic response). In Experiment 2, the significant frequency bands were 0.060-0.065 Hz, which were also in line with the duration of one task trial (i.e., around 15 seconds). The durations of the stimuli in one task trial were different in the two experiments, which was 12 seconds in Experiment 1, but 6 seconds in Experiment 2. We are extremely sorry that we have forgotten to describe this point (we have shortened the duration of the stimuli in Experiment 2 to control the total duration of the experiment). In this way, we think there is a link between the temporal components of the task trials and the significant frequency bands.

Previous fNIRS-hyperscanning studies have showed that the task-related frequencies might correspond with the inverse of the time interval between two continuous trials or task-related events (Cui et al., 2012; Hu et al., 2017; Xue et al., 2018; Yang et al., 2020). Thus, the frequency bands vary across the two experiments because of the different durations of task trials in the experiments.

In the revised paper, we have rewritten the words of the stimuli in Methods of Experiment 2 (line 370-371 in page 15) as follows:

"Stimuli and apparatus. The stimuli were four kinds of meters generated by manipulating the accent (i.e., strong vs. weak) and the frequency of occurrence (i.e., duple vs. triple). For each tone sequence, there were 12 40-Hz pure tones at an interval of ∼500 ms between them. The total duration of the tone sequence for each trial was constant (i.e., 6 s). For the duple meters, every second tone was reduced in intensity by 10 dB or 2 dB, corresponding to strong and weak meters, respectively. For the triple meters, the intensities of every second and third tones were reduced by 10 dB (strong meters) or 2 dB (weak meters). These difference in loudness (i.e., 10 dB vs. 2 dB) have been found in past work to induce significantly different responses (Ozertem & Erdogmus, 2008).”

We have also added the information of the link between the task trials and the significant frequency bands in Results of Experiment 1 (line 342-344 in page 14) and Experiment 2 (line 419-421 in page 17) as follows:

"Enhanced IBS in inter-personal coordination with meter. The cluster-based permutation test identified one channel-frequency cluster that reached significance: the cluster in channel 6 in the range of frequencies of 0.026-0.030 Hz. These frequency bands roughly corresponded to the duration of one task trial in Experiment 1 (i.e., around 33 s based on the participants’ pragmatic response). In this range of frequencies (i.e., 0.026-0.030 Hz), the IBS in channel 6 was significantly greater in the meter condition (0.388 {plus minus} 0.111) than the no-meter condition (0.293 {plus minus} 0.075; Figure 3A). According to the AAL atlas and data of Neurosynth, channel 6 was approximately located in the left-middle frontal cortex.”

"Increased IBS due to accent. The IBS was compared between the conditions (strong vs. weak) by using the paired t-test because we observed a significant effect of the accent on behavioral coordination. The cluster-based permutation test revealed two significant channel-frequency clusters: cluster 1 (frequencies 0.060-0.065 Hz, channel 5) and cluster 2 (frequencies 0.060-0.065 Hz, channel 10). The significant frequency bands were also in line with the duration of a task trial (i.e., around 15 s in Experiment 2).”

As you suggested, the significant IBS in this study might be correlated with the recall of the stimulus, then it would be also found in the independence task. However, such significant IBS was not observed when performing the comparison between the meter-independence condition and no-meter-independence condition by using the paired t-test. The significant IBS was not found when comparing the meter-coordination condition with the meter-independence condition, either. Thus, the revealed IBS might be related to inter-personal coordination with meters. In the updated paper, we have discussed this issue in Discussion (line 515-528 in page 21-22) as follows:

"In this study, we used a beat reproduction task in which the participants were asked to reproduce a sequence of the stimuli after having heard it (Grahn & Brett, 2007). This reproduction task is different from the beat tapping task (e.g., a finger-tapping task in synchrony with a given beat) because the former task also requires a memory component (Steinbrink et al., 2019). The significant IBS reported here might have thus been obtained due to the difference between the memory required for metered/accented tones and that for no-metered tones. However, a significant MFC-IBS was not observed when comparing the meter and no-meter conditions on the independence task by using the paired t-test. In addition, a previous fMRI study showed that the temporal lobe was correlated with the capacity of the auditory working memory during a rhythm reproduction task (Grahn & Schuit, 2012). Thus, the enhanced IBS reported in this study seems to be independent of the memory component. To confirm our findings, future work should explore the significant IBS reported here through the beat tapping task.”

References

Cui, X., Bryant, D. M., & Reiss, A. L. (2012). NIRS-based hyperscanning reveals increased interpersonal coherence in superior frontal cortex during cooperation. NeuroImage, 59(3), 2430-2437.

Hu, Y., Hu, Y., Li, X., Pan, Y., & Cheng, X. (2017). Brain-to-brain synchronization across two persons predicts mutual prosociality. Social Cognitive and Affective Neuroscience, 12(12), 1835-1844.

Xue, H., Lu, K., & Hao, N. (2018). Cooperation makes two less-creative individuals turn into a highly-creative pair. NeuroImage, 172, 527-537.

Yang, J., Zhang, H., Ni, J., De Dreu, C. K., & Ma, Y. (2020). Within-group synchronization in the prefrontal cortex associates with intergroup conflict. Nature Neuroscience, 23(6), 754-760.

As raised in the previous reviews, the trial structure is still unclear:

Line 133 “Each tone sequence lasted for 12 seconds longer, which was composed of 12 tones with an interval of 500-1000 ms between each tone”. Were the tone sequences always 12 seconds? If so, then with shorter inter-stimulus intervals, there would need to be more tones. Conversely, if each trials consisted of 12 tones, then the trial length will vary as a function of the inter-stimulus interval. This should be clarified.

Response: The tone sequences were always 12 seconds. That is, for each tone sequence, the duration is 12 seconds. Although variance of the time interval between tones, the total duration of the tone sequence for each trial was constant (i.e., 12 seconds). We have added the information in the updated manuscript (line 135-136 in page 6) as follows:

"To note, the total duration of the tone sequence for each trial was constant (i.e., 12 seconds).”

Lines 209-212: “We summed...”. This seems to suggest that only trials with interpersonal time lags less than the median were retained. Perhaps I am misunderstanding this but it seems to simply state the smaller interpersonal gaps indicated better synchronization. If this is the data used in subsequent analyses, it could strongly bias the analyses in favor of the hypothesized effect of better metered v. non-metered coordination. It would be helpful to clarify the purpose of this measure and what it is telling us about the data (results ported on lines 297-300).

Response: Yes, smaller inter-personal time lags indicated better synchronization. In Experiment 1, we found a smaller mean inter-personal time lag during the coordination task in the meter condition than the no-meter condition. The probability density of inter-personal time lags was computed to further examine whether the smaller mean inter-personal time lag was due to the variation of distribution of trials with smaller inter-personal time lags in the two experimental conditions. For the estimated probability densities, the probability densities with small inter-personal time lags (i.e., less than the median) were summed and compared between the conditions. To note, all trials were entered into the Kernel Density Estimation. But only the probability densities with small inter-personal time lags were retained in the paired t-test (the regression model in the revision). In the resubmitted paper, we have corrected the words as follows (line 222-229 in page 10):

"The estimated probability densities with short inter-personal time lags (i.e., shorter than the median; 0.4 for our dataset) were summed and compared between the experimental conditions. All trials were entered into the kernel density estimation. However, only probability densities with short inter-personal time lags were retained in the linear mixed model, with the fixed effects of the condition and individual differences as well as the random effect of the dyad. A higher probability density of taps with short inter-personal time lags for dyads also manifested better behavioral coordination.”

Despite reasonably good statistical reporting, there are several areas where more details are needed. In particular, the method of correction for multiple comparisons of the individual subject fNIRS data paired t-tests between the metered and non-metered conditions (channels x frequency bands) suggests that there is a correction for multiple comparisons, but what correction and how it is applied is not described (e.g., lines 273-274). If there is no correction applied, there needs to be.

These comments apply as well to Experiment 2.

Response: In the current work, a cluster-based permutation approach was used to correct for multiple comparisons when analyzing the fNIRS data. The method is a nonparametric technique, rather than familiar correction methods (e.g., Bonferroni, FDR) based on the parametric statistical framework. The cluster-based permutation approach provides a straightforward way to solve the multiple comparisons problem (Maris and Oostenveld, 2007). Nonparametric statistical tests offer complete freedom to use any test statistic one considers appropriate. This freedom allows us to solve the multiple comparisons problem in a simple way. In the revised manuscript, we have tried to make it clear in Methods (line 276-285 in page 12) as follows:

"To determine the frequency band of interest, the linear mixed model was used to compare IBS between experimental conditions across the full range of frequency (0.015-0.1 Hz). The frequencies above 0.1 Hz were removed as they might have been correlated with non-neuronal components, such as Mayer waves (around 0.1 Hz), respiration (0.2-0.6 Hz), and cardiac pulsation (above 0.7 Hz). The frequencies below 0.015 Hz were also removed because IBS has often been observed only at frequencies above 0.015 in fNIRS hyperscanning studies (e.g., Dai et al., 2018; Nguyen et al., 2021; Wang et al., 2019). A cluster-based permutation test was used, in the same manner as in a recent fNIRS hyperscanning study by us (Zhu et al., 2021; Pan et al., 2022). This method is a non-parametric statistical test that allows for multiple comparisons of multi-channel (i.e., 22 channels) and multi-frequency (i.e., 30 frequency bins) data, rather than familiar methods of correction (e.g., Bonferroni, FDR) based on a parametric statistical framework. It provides a straightforward way to solve the problem of multiple comparisons (Maris and Oostenveld, 2007). Non-parametric statistical tests offer complete freedom to use any test statistic one considers appropriate. This allowed us to solve the problem of multiple comparisons in a simple way.”

References

Maris, E., & Oostenveld, R. (2007). Nonparametric statistical testing of EEG-and MEG-data. Journal of Neuroscience Methods, 164(1), 177-190.

Line 288-289: the reported statistics are not identified (mean {plus minus} sem?)

The purpose of Figure 2B is unclear. It seems wholly descriptive and the meaning of the boxed region is poorly explained.

Response: The reported statistics are mean and deviation. In the revision, we have tried to make it clear (line 307-308 in page 13) as follows:

"Behavioral coordination. The results of the linear mixed model showed that during the coordination task, the mean inter-personal time lag in the meter condition (mean {plus minus} deviation: 0.379 {plus minus} 0.071) was significantly shorter than in the no-meter condition (0.408 {plus minus} 0.060, F = 5.086, p = 0.037, β = -0.231, SE = 0.094, 95% confidence interval (CI) = -0.399 to -0.028; Figure 2A).”

The purpose of Figure 2B was to show that the probability densities with small inter-personal time lags between conditions were insignificantly different. We have changed Figure 2B as follows in the modified paper:

"Figure 2. Behavioral performance on the coordination task between the meter and no-meter conditions in Experiment 1. (A) Mean inter-personal time lag (Mean lag). Shorter mean inter-personal time lag in the meter condition compared with that in the no-meter condition. (B) Probability density estimation of all inter-personal time lags (Lag). There was no significant difference in the probability densities of short inter-personal time lag (shorter than the median; the dashed box) between the meter and no-meter conditions. (C) Hurst exponent H of detrended fluctuation analysis. There was no significant difference in H between the meter and the no-meter conditions. (D) Linear regression of inter-personal time lags across taps in the meter and no-meter conditions. The inter-personal time lags significantly decreased across taps in the meter condition. Boxplots are presented, with the horizontal lines indicating the median values, boxes indicating 25% and 75% quartiles, and error bars representing minimum/maximum values. The diamond dots represent extreme values. *p < 0.05, n.s. not significant.”

Although the mean lags are smaller for metered than non-metered stimuli, the variance (or at least the range) for the metered conditions is larger than the non-metered condition (line 288 and Fig 2a). This is counter-intuitive and suggests there is greater variability among the dyads in the metered condition.

Response: The deviations of the two conditions were 0.071 and 0.060, respectively. In Figure 2A, data were plotted by boxplots with horizontal lines indicating median values, boxes indicating 25% and 75% quartiles, and error bars represented minimum/ maximum values. According to Figure 2A, the minimum mean inter-personal time lag in the meter condition was intuitively lower than that in the no-meter condition. Specifically, the mean inter-personal time lags varied from 0.209 to 0.507 in the meter condition, while they varied from 0.330 to 0.563. We have tried to make it clear as follows in the caption of Figure 2 as follows:

"Figure 2. Behavioral performance on the coordination task between the meter and no-meter conditions in Experiment 1. (A) Mean inter-personal time lag (Mean lag). Shorter mean inter-personal time lag in the meter condition compared with that in the no-meter condition. (B) Probability density estimation of all inter-personal time lags (Lag). There was no significant difference in the probability densities of short inter-personal time lag (shorter than the median; the dashed box) between the meter and no-meter conditions. (C) Hurst exponent H of detrended fluctuation analysis. There was no significant difference in H between the meter and the no-meter conditions. (D) Linear regression of inter-personal time lags across taps in the meter and no-meter conditions. The inter-personal time lags significantly decreased across taps in the meter condition. Boxplots are presented, with the horizontal lines indicating the median values, boxes indicating 25% and 75% quartiles, and error bars representing minimum/maximum values. The diamond dots represent extreme values. *p < 0.05, n.s. not significant.”

The design of the experiments really tests whether the memory for metered and accented tones are more effective in synchronizing behavior than memory for non-metered tones, because as I understand it, the subject repeats a sequence after hearing it. This distinction between a remembered and a present rhythm is important because in the current case it is not the “music” per se that is driving the coordination between individuals, but the ability of the individuals to accurately retain the rhythm.

Response: We agree that there is difference between a remembered and a present rhythm. In the current work, participants were asked to reproduce a sequence of the auditory stimuli after hearing it. This is in fact a beat reproduction task (Grahn and Brett, 2007). Beat tapping might be another task, such as a finger tapping task in synchrony with a present beat. The two tasks both require listening and motor coordination. The reproduction task also requires working memory and grouping events in meaningful chunks, even though the sequences were not long. But the tapping task is a sensorimotor synchronization task which does not require working memory or chunking because the stimulus was present (Steinbrink et al., 2019). Thus, for the dependence task in this study, there might be two key processes: auditory-motor coordination and working memory. For the coordination task, there might be three processes: auditory-motor coordination, working memory, and mutual coordination with the partner.

As you suggested, the memory component may play a role in the current work. Our findings may be due to the difference between the memory for metered/accented tones and the memory for no-metered tones. However, the significant IBS reported here was not observed when compared the meter and no-meter conditions on the independence task by using the paired t-test. In addition, a previous fMRI study reported that the activity in posterior superior temporal gyms and middle temporal gyms negatively correlated with auditory working memory capacity (i.e., the amount of auditory information that can be remembered over a few seconds) during the rhythm reproduction task (Grahn & Schuit, 2012). The previous findings suggest that the temporal region plays an important role in working-memory process during the beat reproduction task. In this study, the enhanced IBS is located at the middle frontal cortex, which seems to be independent of working memory. To confirm our findings, future study can further explore the significant IBS reported here by using the beat tapping task.

In the updated article, we have discussed this issue in Discussion as follows (line 515-528 in page 22):

"In this study, we used a beat reproduction task in which the participants were asked to reproduce a sequence of the stimuli after having heard it (Grahn & Brett, 2007). This reproduction task is different from the beat tapping task (e.g., a finger-tapping task in synchrony with a given beat) because the former task also requires a memory component (Steinbrink et al., 2019). The significant IBS reported here might have thus been obtained due to the difference between the memory required for metered/accented tones and that for no-metered tones. However, a significant MFC-IBS was not observed when comparing the meter and no-meter conditions on the independence task by using the paired t-test. In addition, a previous fMRI study showed that the temporal lobe was correlated with the capacity of the auditory working memory during a rhythm reproduction task (Grahn & Schuit, 2012). Thus, the enhanced IBS reported in this study seems to be independent of the memory component. To confirm our findings, future work should explore the significant IBS reported here through the beat tapping task.”

References

Grahn, J. A., & Brett, M. (2007). Rhythm and beat perception in motor areas of the brain. Journal of cognitive neuroscience, 19(5), 893-906.

Grahn, J. A., & Schuit, D. (2012). Individual differences in rhythmic ability: Behavioral and neuroimaging investigations. Psychomusicology: Music, Mind, and Brain, 22(2), 105.

Steinbrink, C., Knigge, J., Mannhaupt, G., Sallat, S., & Werkle, A. (2019). Are Temporal and tonal musical skills related to phonological awareness and literacy skills?-Evidence from two cross-sectional studies with children from different age groups. Frontiers in psychology, 10, 805.

The error bars and shaded regions of the bar charts need to be defined.

Response: Done as suggested. Please find the added information as follows in the revised paper as follows:

"Figure 2. Behavioral performance on the coordination task between the meter and no-meter conditions in Experiment 1. (A) Mean inter-personal time lag (Mean lag). Shorter mean inter-personal time lag in the meter condition compared with that in the no-meter condition. (B) Probability density estimation of all inter-personal time lags (Lag). There was no significant difference in the probability densities of short inter-personal time lag (shorter than the median; the dashed box) between the meter and no-meter conditions. (C) Hurst exponent H of detrended fluctuation analysis. There was no significant difference in H between the meter and the no-meter conditions. (D) Linear regression of inter-personal time lags across taps in the meter and no-meter conditions. The inter-personal time lags significantly decreased across taps in the meter condition. Boxplots are presented, with the horizontal lines indicating the median values, boxes indicating 25% and 75% quartiles, and error bars representing minimum/maximum values. The diamond dots represent extreme values. *p < 0.05, n.s. not significant.”

While the writing is generally good, there are some grammatical and syntactic errors that make the manuscript difficult to understand, and it would benefit from editing by a fluent English speaker.

Response: Done as suggested. We have used a language-editing service to improve the writing of the updated manuscript.
